# Relation of retinal and hippocampal thickness in patients with amnestic mild cognitive impairment and healthy controls

**DOI:** 10.1002/brb3.2035

**Published:** 2021-01-15

**Authors:** Markus Donix, Dierk Wittig, Wiebke Hermann, Robert Haussmann, Maren Dittmer, Franziska Bienert, Maria Buthut, Liane Jacobi, Annett Werner, Jennifer Linn, Tjalf Ziemssen, Moritz D. Brandt

**Affiliations:** ^1^ Department of Psychiatry University Hospital Carl Gustav Carus Technische Universität Dresden Dresden Germany; ^2^ German Center for Neurodegenerative Diseases (DZNE) Dresden Germany; ^3^ Department of Ophthalmology University Hospital Carl Gustav Carus Technische Universität Dresden Dresden Germany; ^4^ Department of Neurology University Hospital Rostock Germany; ^5^ German Center for Neurodegenerative Diseases (DZNE) Rostock Germany; ^6^ Department of Neurology Sächsisches Krankenhaus Arnsdorf Arnsdorf Germany; ^7^ Department of Neuroradiology University Hospital Carl Gustav Carus Technische Universität Dresden Dresden Germany; ^8^ Department of Neurology University Hospital Carl Gustav Carus Technische Universität Dresden Dresden Germany

**Keywords:** hippocampus, magnetic resonance imaging, mild cognitive impairment, optical coherence tomography, retina

## Abstract

**Objective:**

Investigating retinal thickness may complement existing biological markers for dementia and other neurodegenerative diseases. Although retinal thinning is predictive for cognitive decline, it remains to be investigated if and how this feature aligns with neurodegeneration elsewhere in the brain, specifically in early disease stages.

**Methods:**

Using optical coherence tomography and magnetic resonance imaging, we examined retinal thickness as well as hippocampal structure in patients with amnestic mild cognitive impairment and healthy controls.

**Results:**

The groups did not differ in hippocampal and retinal thickness measures. However, we detected a correlation of peripapillary retinal nerve fiber layer thickness and hippocampal thickness in healthy people but not in cognitively impaired patients. The ratio of hippocampus to retina thickness was significantly smaller in patients with mild cognitive impairment and correlated positively with cognitive performance.

**Conclusions:**

Different temporal trajectories of neurodegeneration may disrupt transregional brain structure associations in patients with amnestic mild cognitive impairment.

## Graphical abstract

We investigated in patients with mild cognitive impairment, if and how retinal thinning aligns with neurodegeneration elsewhere in the brain. We detected a correlation of peripapillary retinal nerve fiber layer thickness and hippocampal thickness in healthy control participants but not in the cognitively impaired individuals. Different temporal trajectories of neurodegeneration may disrupt transregional brain structure associations in early neurodegeneration.

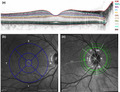

## 1 INTRODUCTION

Patients with amnestic mild cognitive impairment (MCI) are at high risk for developing dementia, mainly Alzheimer's disease with up to 90% of patients affected in clinical trials (Petersen, 2011; Petersen et al., 2005). They suffer from subjective and measurable memory impairment that does not substantially interfere with daily functioning thus not fulfilling criteria for dementia. However, current research is focusing on establishing biomarkers to predict further cognitive decline and risk of conversion to dementia. Hippocampal atrophy detected with magnetic resonance imaging (MRI) is a predictor for cognitive decline among patients with amnestic MCI (Risacher et al., 2009). The dynamic pattern of gray matter atrophy over time in Alzheimer's disease (Thompson et al., 2003) also corresponds well to the clinical syndrome once a patient or others become aware of the cognitive changes. This pattern of atrophy reflects how Alzheimer's disease's neuropathology—amyloid plaques and neurofibrillary tangles—spread throughout the brain. On the molecular level, neurofibrillary tangle deposition resembles this pattern more closely than amyloid (Braak & Braak, 1991). Although Alzheimer's disease is considered a progressive neurodegenerative process with dementia evolving as the final stage of the disease, neither hippocampal structure assessments nor cerebrospinal fluid (CSF) analyses of amyloid and tau pathology are yet sufficient biomarkers to confirm definitive future development of dementia in patients with MCI.

In the search for new biological markers, investigating the eye with optical coherence tomography (OCT) (Coppola et al., 2015) has become a method for integrating additional information about degenerative brain changes in vivo. The retina can be examined noninvasively using the optical imaging procedure, and the technique's precision allows detecting changes in macula volume and thickness as well as thickness of the peripapillary retinal nerve fiber layer (pRNFL, the retina's inner layer consisting of retinal ganglion cell axons) across subregions of the structures' anatomical perimeters. Since Scinto et al. (1994) reported an altered pupil dilatation response to cholinergic eye drops among Alzheimer's disease patients, there is converging evidence for potential ocular biomarkers in Alzheimer's disease (Chan et al., 2019; Frost et al., 2010; Javaid et al., 2016). When structural abnormalities were detected examining pRNFL images in Alzheimer's disease patients (Hedges et al., 1996), animal models of the disease and human postmortem data later revealed the presence of beta amyloid depositions in the retina (Koronyo‐Hamaoui et al., 2011). Mutlu et al. (2018) showed that pRNFL thinning is associated with increased risk of developing Alzheimer's disease in a large prospective population‐based study. pRNFL thinning is associated with elevated Alzheimer's disease CSF biomarkers in cognitively healthy individuals (Asanad et al., 2020) and predicts future cognitive decline in people without a neurodegenerative disease (Ko et al., 2018). Chan and colleagues highlight the potential of OCT to obtain new Alzheimer's disease biomarkers. In their meta‐analysis, investigating patients with MCI and Alzheimer's disease, they found an association of several OCT measurements including peripapillary RNFL thinning in patients with Alzheimer's disease (Chan et al., 2019). The authors also analyzed 6 studies that focused on RNFL thickness among MCI patients and controls. There was a trend of RNFL thinning in MCI patients, although not statistically significant (Chan et al., 2019).

When investigating Alzheimer's disease biomarkers, it should be further determined how pRNFL thinning aligns with or complements other measures of neurodegeneration. Detecting neurodegeneration‐associated changes in medial temporal lobe structure in patients with MCI can be challenging using MRI. Therefore, we did not investigate hippocampal volume but hippocampal thickness, using an advanced image segmentation and data analysis approach within the convoluted but functionally heterogeneous structures of the medial temporal lobe (Zeineh, Engel, Thompson, & Bookheimer, 2001, 2003). We previously demonstrated that with this technique we could identify characteristics of radiological anatomy even among small samples of yet healthy older individuals at risk for developing dementia (Baumgaertel et al., 2016; Donix et al., 2010, 2013).

Although markers of retinal neurodegeneration show an association with cerebral atrophy in a population‐based study across the whole brain (Mutlu et al., 2017), the underlying mechanisms and dynamics of such associations remain largely unknown. Santos et al. (2018) revealed that in patients suffering from preclinical Alzheimer's disease, regional macular RNFL volume was linearly related to neocortical amyloid deposition using positron emission tomography. Others suggest that pRNFL thickness may undergo thinning and thickening during Alzheimer's disease progression (Lad et al., 2018). Using OCT and MRI as well as thickness measurements within the retina and the hippocampus, we hypothesized that amnestic MCI patients would show a thinner pRNFL and reduced hippocampal thickness when compared with healthy older people. Because pRNFL thinning and hippocampal thinning likely follow different temporal gradients in neurodegeneration, the ratio of pRNFL thickness and hippocampal thickness would change with disease progression. We therefore hypothesized that this ratio would be smaller among patients with MCI when compared with healthy participants and would correlate with functional impairment.

## 2 METHODS

### 2.1 Participants

Twenty‐three people, recruited through our university hospital's memory clinic, participated in this pilot study after obtaining written informed consent. The study was conducted in accordance to the ethical principles of the Declaration of Helsinki (ICH) and the university's ethics committee approved the study (EK 363082015). We included 12 healthy controls (mean age 65.1 years ± 9.0 years) and 11 individuals with amnestic MCI (mean age 68.6 years ± 6.7 years). All subjects were identified from a subject sample recruited during 2016 and 2017. MCI patients met standard diagnostic criteria (Petersen, 2011). These criteria are memory complaints and a measurable memory impairment below age norms (scoring at least 1 standard deviation below age‐ and education‐adjusted norms in the word list recall subtest of the Consortium to Establish a Register for Alzheimer's Disease‐Neuropsychological (CERAD‐NP) test battery), which does not (yet) interfere with daily functioning. The CERAD‐NP is one of the most widely used neurocognitive test batteries for a detailed assessment of pathological cognitive dysfunction (Morris et al., 1989). It was specifically designed to detect early changes in the Alzheimer's disease spectrum, for example subtle memory deficits. The CERAD‐NP consists of eight subtests across different cognitive domains. The subtests are for the examination of verbal fluency (Animal Naming Test), word finding (modified Boston Naming Test), general cognitive function (Mini‐Mental‐Status‐Test), verbal memory (word list learning, delayed recall, and recognition), visual memory, and visuoconstructive abilities. Although there are approaches to establish a total score (not including the MMSE screening test) (Chandler et al., 2005), age‐adjusted norms for the subtest scores are used as a standard in neurocognitive examinations.

The clinical procedures to rule out dementia and establish the diagnosis of amnestic MCI, which themselves were not part of this study, include a clinical interview and examination by a physician, neurocognitive testing across cognitive domains as outlined above, laboratory testing and cerebral imaging to detect conditions, such as thyroid dysfunction or cerebrovascular disease, that would explain the cognitive deficit otherwise. The study participants did not have a psychiatric or neurological disorder other than amnestic MCI and did not suffer from a systemic disease affecting brain functioning. All study participants were right‐handed (self‐report) and did not receive sedating or stimulating psychotropic medication. Two control participants did not participate in MRI scanning, and two individuals (one patient and one control subject) did not receive OCT measurements due to glaucoma and macular degeneration, which represented specific exclusion criteria.

### 2.2 Procedures

**FIGURE 1:**
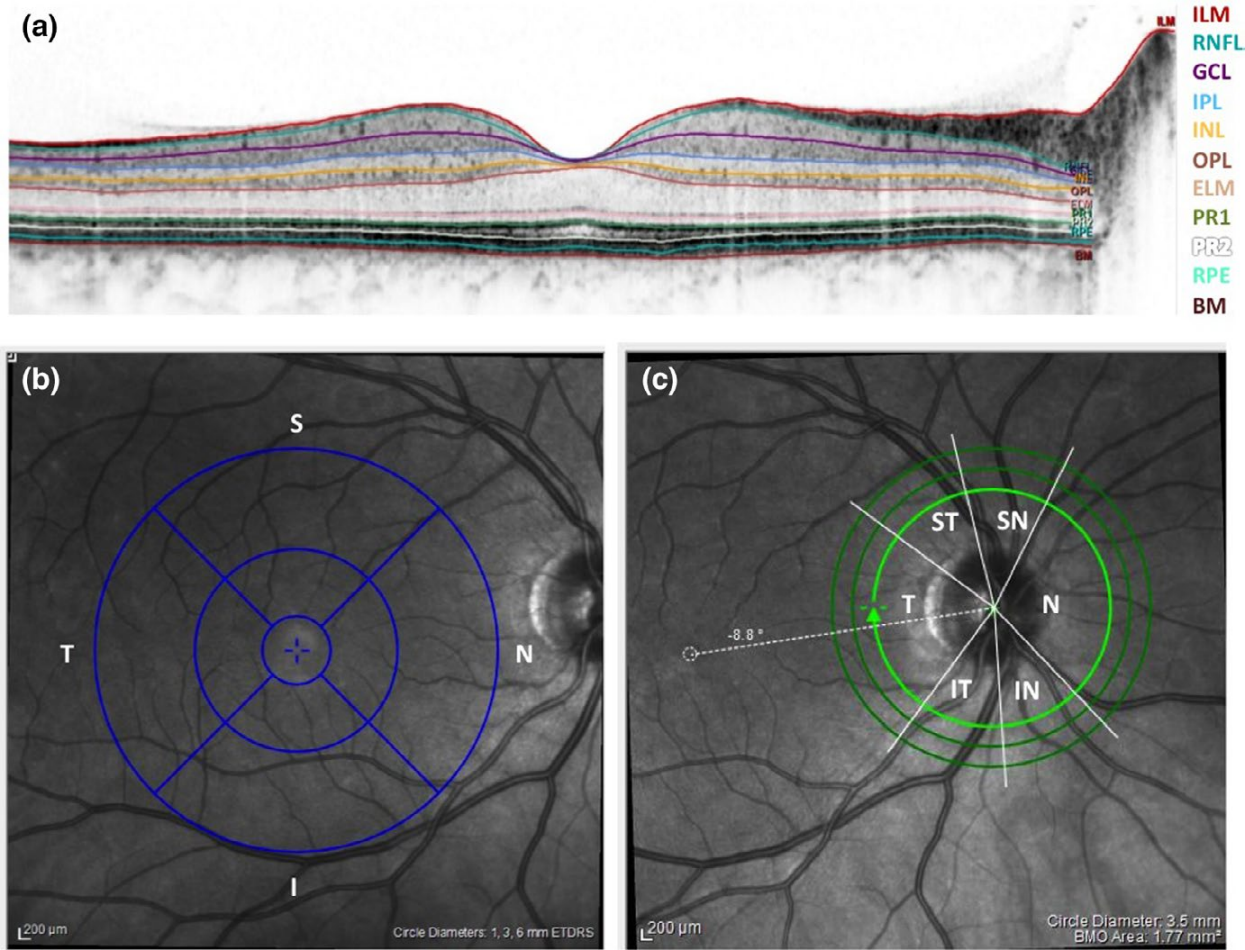
OCT measurements and retinal layers. Visualization of the laminar structure of the retina (a), as well as regions of interest for macular (b) and peripapillary retina nerve fiber layer (c) thickness. R = right, L = left, T = temporal, S = superior, *N* = nasal, I = inferior, ILM = internal limiting membrane, NFL = nerve fiber layer, GCL = ganglion cell layer, IPL = inner plexiform layer, INL = inner nuclear layer, OPL = outer plexiform layer, ONL = outer nuclear layer, ELM = external limiting membrane

With a Spectralis OCT device and segmentation software (Heidelberg Engineering GmbH, Heidelberg, Germany) we administered a 12° circular scan of the optic nerve head (B‐scans: 3, ART[AutomaticRealTime]: 100) and a macular scan with a diameter of at least 6 mm (B‐scans: 49, scan size 25° × 30°, ART: 15) to investigate peripapillary pRNFL thickness and macular thickness across all retinal layers (Figure 1). In this pilot study, we specifically focused on pRNFL thickness. Due to left eye glaucoma in two participants and missing pRNFL data in a third individual, we restricted our analyses on the complete dataset of the right eye.

Using a Siemens Magnetom Verio 3‐Tesla scanner (Siemens Medical Solutions, Erlangen, Germany) with a 12 channel head coil (receive only), we obtained high‐resolution oblique coronal T2‐weighted fast‐spin echo scans (repetition time: 5,200 ms; echo time: 105 ms; slice thickness: 3 mm; spacing: 0 mm; 19 slices; in‐plane voxel size: 0.39 × 0.39 mm; field of view: 200 mm) and T1‐weighted magnetization‐prepared rapid acquisition gradient‐echo (MPRAGE) scans (repetition time 2,300 ms, echo time 2.93 ms, voxel size 1 mm^3^, 160 slices, FOV 256 mm). We used T1 data and the FreeSurfer toolkit (http://www.surfer.nmr.mgh.harvard.edu) for cortical surface reconstruction and cortical thickness measurements (Dale et al., 1999; Fischl & Dale, 2000; Fischl et al., 1999). After anatomical atlas (Destrieux et al., 2010) guided cortical parcellation (Fischl et al., 2004), thickness maps are created using spatial intensity gradients across tissue classes (Fischl & Dale, 2000). Thickness calculations have been validated by histological and manual measurements (Han et al., 2006; Rosas et al., 2002).

The T2‐weighted data were subjected to a cortical unfolding‐based analysis (Zeineh et al., 2001, 2003). This technique flattens the medial temporal lobe cortex into two‐dimensional space, enabling regional thickness measurements of its small and convoluted structures. After manually masking white matter and CSF on the original MRI sequence, the remaining gray matter volume covers the cornu ammonis fields 1–3 and the dentate gyrus, the subiculum, entorhinal, perirhinal and parahippocampal cortices, and the fusiform gyrus. Hippocampal thickness was defined as average thickness of the cornu ammonis fields including the dentate gyrus, and the subiculum. Cortical thickness measurements are performed after computational unfolding and flattening of this volume with metric multidimensional scaling (Zeineh et al., 2001, 2003). Boundaries between the regions are delineated using histological and MRI atlases (Amaral & Insausti, 1990; Duvernoy, 1998), before they are mathematically projected to the two‐dimensional space.

Within the FreeSurfer environment, we utilized a general linear model, and the 'Query, Design, Execute, Contrast' (QDEC) application to investigate the influence of pRNFL thickness on cortical thickness across the whole brain (false discovery rate correction for multiple comparisons, *p* < .05). Group comparison of pRNFL thickness was performed utilizing univariate analysis of variance. We investigated hippocampal thickness using multivariate analyses of variance with right or left hippocampal thickness measures as the dependent variables and diagnosis (amnestic MCI/control) as between‐group factor. In order to reveal possible associations between regional retinal thickness and hippocampal thickness within both hemispheres we utilized Pearson correlation analyses. As a safeguard against spurious findings, we only examined regional pRNFL thickness/hippocampal thickness correlations and ratios after establishing significance between hippocampal thickness and mean global pRNFL thickness. We calculated thickness ratios only in the regions with significant pRNFL thickness/hippocampal thickness correlations and subsequently correlated the ratios to cognitive function. We controlled for age in all analyses, and we used a significance level of *p* < .05, two‐sided. Gender distribution was compared using the chi‐square test.

## 3 RESULTS

**FIGURE 2:**
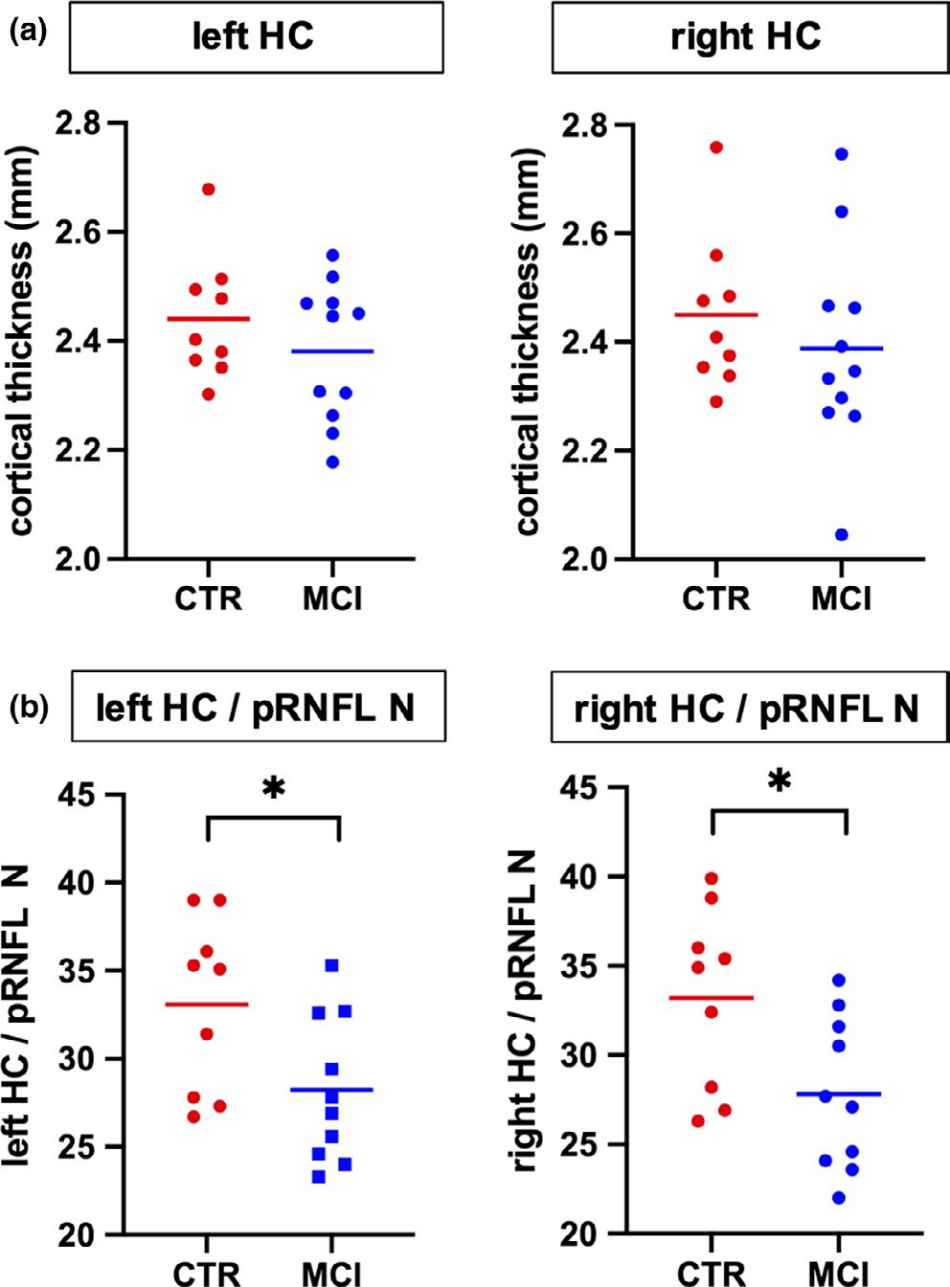
Group comparison of hippocampal thickness and hippocampal to retinal thickness ratio. Between‐group comparisons revealed no difference in hippocampal thickness (a), but a significant lower ratio of hippocampal to retinal thickness in MCI patients (b). HC = hippocampus, pRNFL N = nasal peripapillary retina nerve fiber layer, MCI = mild cognitive impairment, CTR = healthy controls

**FIGURE 3:**
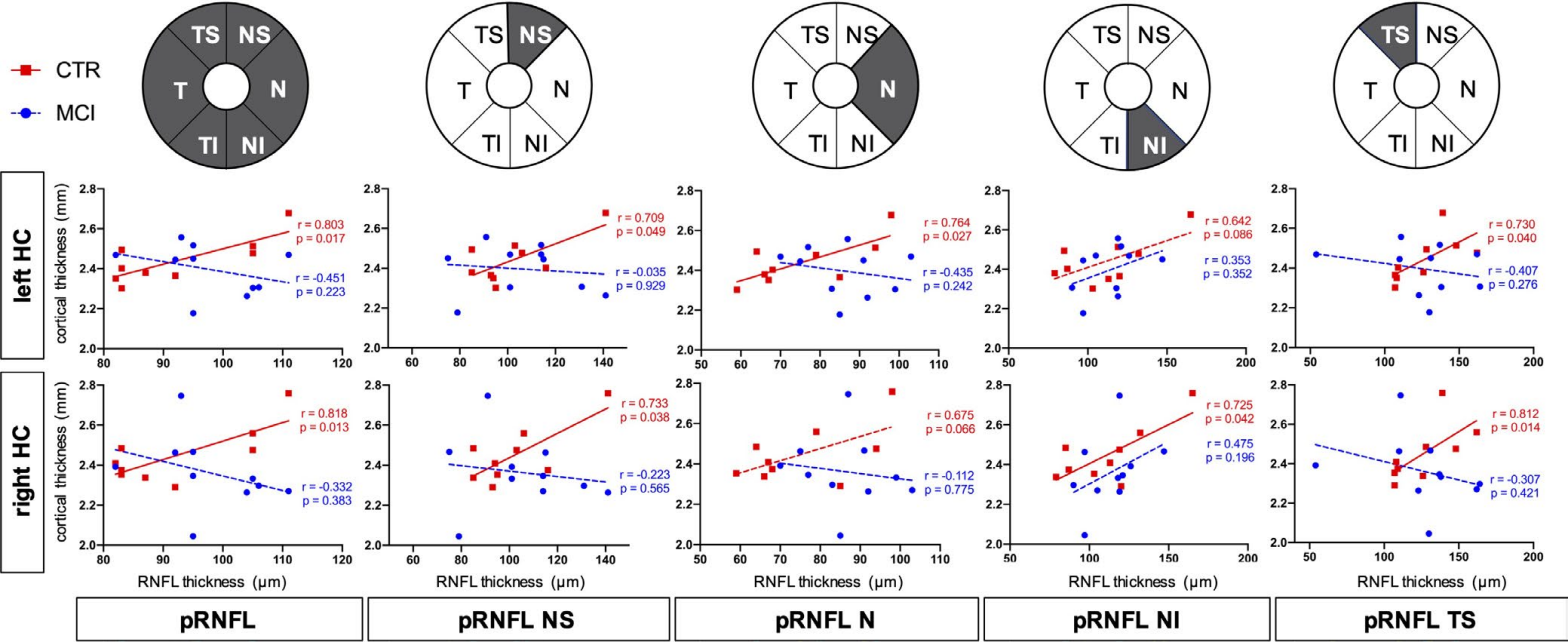
Associations of pRNFL thickness and hippocampal thickness. There is a positive correlation (highlighted subregions) of hippocampal thickness and pRNFL thickness in healthy controls (CTR) but not in subjects with amnestic mild cognitive impairment (MCI). HC = hippocampus, pRNFL = peripapillary retina nerve fiber layer, MCI = mild cognitive impairment, T = temporal, S = superior, *N* = nasal, I = inferior

**FIGURE 4:**
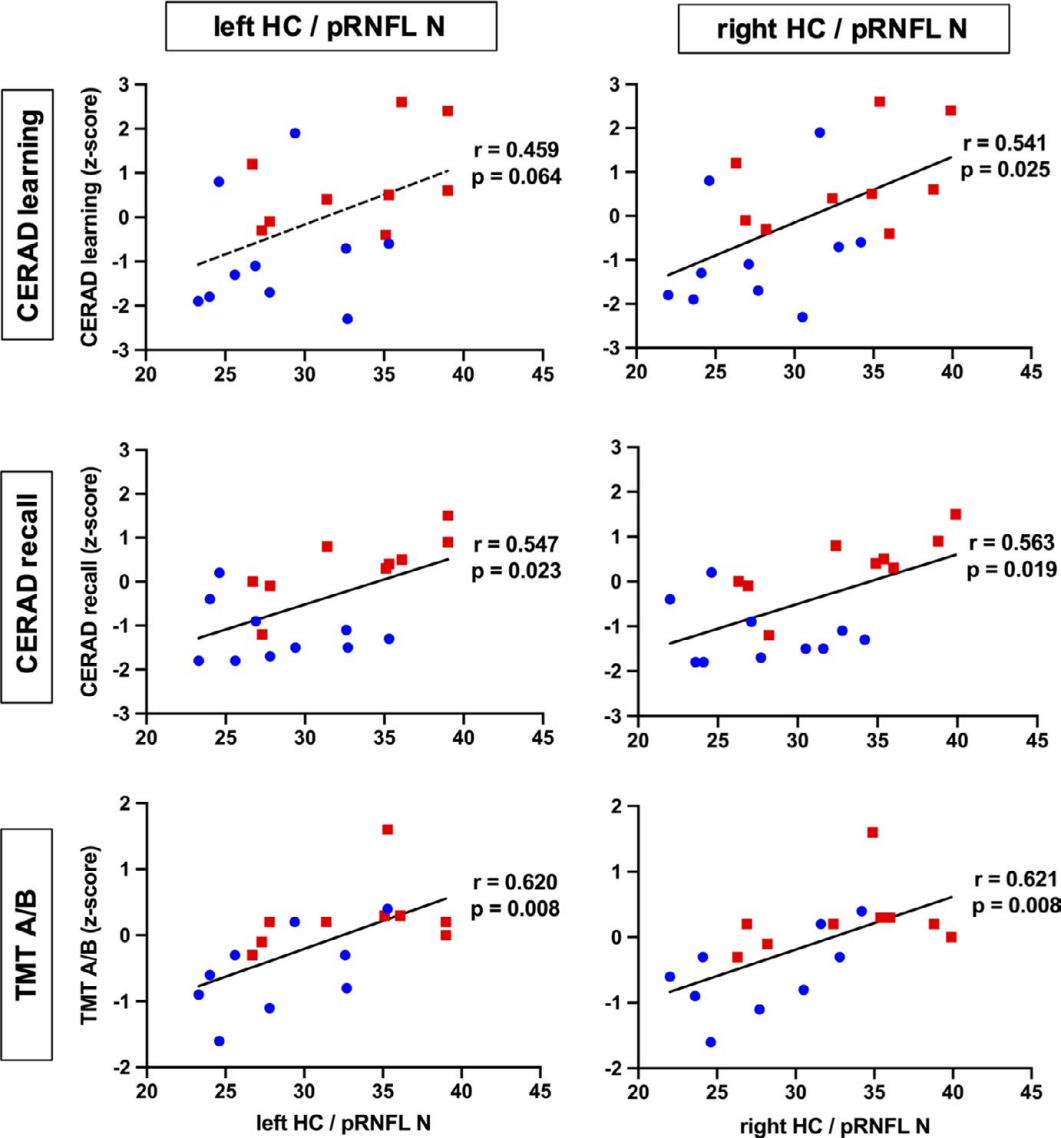
Age‐adjusted correlation of hippocampal to retinal thickness ratio with cognitive performance. Ratio of hippocampal to nasal retinal thickness correlates with CERAD memory and trail making test performance. HC = hippocampus, pRNFL *N* = nasal peripapillary retina nerve fiber layer, CERAD = Consortium to Establish a Registry for Alzheimer's Disease neurocognitive test battery, TMT A/B = trail making test, MCI = mild cognitive impairment, CTR = healthy controls

Our study participants did not significantly differ in mean age, gender distribution, or educational status (Table 1). In the hippocampal and whole brain cortical thickness analysis, thickness did not differ between the groups at the predefined statistical threshold (Figure 2a). Although the groups also did not differ in average pRNFL thickness, we found positive correlations between hippocampal thickness and regional, predominantly nasal and superior pRNFL thickness (Figure 3). These age‐adjusted correlations were detectable only among control participants, but not in MCI patients. Left hemispheric hippocampal thickness correlated with mean global pRNFL thickness (Pearson's *r* = .8, *p* = .017). Subsequent analyses of retinal subregions revealed significant correlations in the following areas: superior temporal region (Pearson's *r* = .73, *p* = .04), nasal region (*r* = .76, *p* = .027), and superior nasal region (*r* = .71, *p* = .049). Right hemispheric hippocampal thickness correlated with mean global pRNFL thickness (Pearson's *r* = .82, *p* = .013), and the corresponding subregions: superior temporal region (Pearson's *r* = .81, *p* = .014), superior nasal region (*r* = .73, *p* = .038), and inferior nasal region (*r* = .73, *p* = .042). The ratio of hippocampal thickness and nasal pRNFL thickness was significantly smaller in MCI patients (left hippocampus/pRNFL N: *F* = .794, *p* = .031; right hippocampus/pRNFL N: *F* = .434, *p* = .022; Figure 2b). The ratio of hippocampal thickness and other than nasal regional pRNFL thickness measures did not significantly differ between MCI patients and control subjects. Taking hippocampal function into account, hippocampal thickness to nasal pRNFL thickness ratio correlated positively with memory performance and executive function in the CERAD: wordlist learning and recall, and in the trail making test (Figure 4, Table 2).

**TABLE 1:** Demographic and clinical characteristics

Characteristics and Measures	CTR	*SD*	MCI	*SD*	Significance (*p*‐value)^a^
*N*	12		11		
Age (years)	65.1	±9.0	68.6	±6.7	.14
Female sex (*N*)	5		5		.85
Education (years)	16.3	±2.3	16.5	±2.7	.45
Mean pRNFL thickness (μm)	90.6	±13.2	97.8	±8.1	.09
Left hippocampal thickness (mm)	2.4	±0.2	2.4	±0.1	.41
Right hippocampal thickness (mm)	2.4	±0.2	2.4	±0.2	.36

Abbreviations: CTR, control participants; MCI, mild cognitive impairment; pRNFL, peripapillary retinal nerve fiber layer; *SD*, standard deviation.

^a^ Chi‐square tests for gender distribution.

**TABLE 2:** Neurocognitive data

Neurocognitive data	CTR	*SD*	MCI	*SD*	Significance (*p*‐value)
MMSE (score range 0–30)	29.3	±0.7	27.7	±2.0	.01
CERAD word list learning (0–10)	8.8	±0.9	7.3	±1.6	.02
CERAD word list learning (z‐score)	0.8		−0.9		.01
CERAD word list recall (0–10)	8.3	±1.3	5.2	±1.8	<.001
CERAD word list recall (z‐score)	0.3		−1.2		<.001
TMT A (sec)	35.8	±11.5	60.4	±39.1	.06
TMT B (sec)	69.1	±17.1	128.1	±46.6	<.001
TMT A/B (z‐score)	0.3		−0.6		.01

Abbreviations: CERAD‐NP, Consortium to Establish a Register for Alzheimer's Disease‐Neuropsychological test battery; CTR, control participants; MCI, mild cognitive impairment; MMSE, Mini‐Mental State Examination; *SD*, standard deviation; TMT A/B, Trail Making Test part A and B quotient; z‐score, standard deviations from the mean age‐ and education‐adjusted norms.

## 4 DISCUSSION

We show that patients with amnestic MCI and cognitively healthy participants do not differ in hippocampal and retinal thickness. However, there are heterogeneous data on retinal thinning in people with cognitive decline (Coppola et al., 2015; den Haan et al., 2018; Paquet et al., 2007). We demonstrate a positive correlation of left and right hippocampal thickness and pRNFL thickness in healthy participants, but not among patients with amnestic MCI. The small hemispheric differences in hippocampal / pRNFL thickness associations across retinal subregions and respective ratios could reflect an asymmetric distribution of neurodegenerative changes (Thompson et al., 2003). Our findings are in line with data from Casaletto and colleagues, who previously revealed an association of medial temporal lobe atrophy and retinal thinning in cognitively healthy older adults (Casaletto et al., 2017).

In patients with dementia, damage to brain regions comprising the visual tract may cause retrograde degeneration of the optic nerve, manifesting as pRNFL thinning (Mutlu et al., 2018). However, among healthy people, the correlation of retinal and cortical thickness could reflect general transregional associations of brain structure and neuronal networks (Rolls et al., 2008). It is less likely that such a correlation indicates pathology, although it cannot be ruled out using cross‐sectional neuroimaging data (Casaletto et al., 2017). In line with this perception is a recent study by Shi and colleagues (Shi et al., 2020). The authors showed an association of pRNFL thickness and hippocampal volume among nondemented older adults. However, if the correlation between pRNFL thickness and hippocampal thickness would already indicate pathology in cognitively healthy individuals, a retrograde optic nerve degeneration mechanism (Mutlu et al., 2018) is not plausible. Furthermore, there is no evidence for a concurrent temporal gradient underlying onset and progression of neurodegeneration in ocular and hippocampal regions. The absence of retina/cortex thickness correlations in patients with amnestic MCI but not in healthy people could therefore reflect a disintegration of regional brain networks and structures in MCI patients due to neurodegenerative changes. Our hypothesis is further facilitated by Liu and colleagues, which demonstrate a disruption of associations between retinal and brain structure across gray and white matter areas due to neurodegeneration (Liu et al., 2016). The primarily nasal and superior localization of pRNFL thickness correlating with hippocampal thickness could be interpreted in this context. There are variable data on whether there is general (Iseri et al., 2006; Parisi et al., 2001) or predominantly superior (Berisha et al., 2007; Kwon et al., 2017; Lad et al., 2018) pRNFL thinning in MCI and Alzheimer's disease. The latter would correspond to the frequently observed vision loss in the inferior visual field in these individuals (Frost et al., 2010; Trick et al., 1995).

Neurodegenerative changes in posterior brain regions involved in visual processing occur relatively early in Alzheimer's disease development. However, neurofibrillary tangle and beta amyloid depositions in this area do not correlate with medial temporal pathology in postmortem brain samples (McKee et al., 2006). Alzheimer's disease's pathological hallmarks have been found in retinal specimen in animal models of the disease (Grimaldi et al., 2018), but there are contrasting data with respect to their presence in the human retina (Kusne et al., 2017). In addition, pRNFL thinning has been reported in frontotemporal dementia (Ferrari et al., 2017), as well as in Parkinson's disease and dementia with Lewy bodies (Hajee et al., 2009; Moreno‐Ramos et al., 2013). Considering the differences of the diseases' underlying neuropathology, a possible association with retinal structure changes may vary in it' predictive value for the temporospatial gradient of neurodegeneration elsewhere in the brain. Our data suggest that in the prodromal stage of Alzheimer´s disease structural changes within the hippocampus may precede those in the retina and thereby abolish a physiological association of cortical and retinal thickness (den Haan et al., 2018; Shi et al., 2020). However, we did not find a significant difference of hippocampal thickness between MCI patients and healthy controls. It is possible that a subtle regional atrophy does not exceed intraindividual variance in our small subject sample. Focusing on the ratio of hippocampal to retinal thickness we essentially normalize hippocampal thickness individually, which indicates disproportional hippocampal atrophy in relation to retina thinning among patients with MCI. The ratio of hippocampal thickness to nasal pRNFL thickness was significantly decreased in MCI subjects compared to healthy controls. Therefore, the relative thinning of the hippocampus in relation to individual pRNFL thickness could be a sensitive marker for regional neurodegeneration. Individual normalization of hippocampal thickness to other brain regions like the retina might therefore be a more accurate approach to assess local neurodegeneration in prodromal Alzheimer´s disease. The hippocampus to pRNFL ratio correlated positively with cognitive function, suggesting clinical relevance of this morphological readout. Study limitations include the cross‐sectional design that allows no direct insight into temporality, incomplete datasets among select individuals as mentioned in the methods section, and the limited sample size, reducing statistical power. Other biological markers derived from CSF and positron emission tomography data should be integrated in longitudinal assessments of people at risk for neurodegeneration. There is an incremental value of biological marker combinations (Frolich et al., 2017), and measuring retinal thickness with OCT will likely complement established technological and clinical assessments for the prediction of cognitive decline and dementia (Ko et al., 2018; Mutlu et al., 2018). Clinicians should also consider the limitations of OCT in the etiological differentiation and staging of cognitive impairment, as well as the frequency of ocular pathology in older people that may interfere with OCT.

## 5 CONCLUSIONS

In contrast to healthy subjects, retinal and hippocampal thickness do not correlate in patients with MCI. This suggests that irrespective of retinal or hippocampal thinning, the missing correlation between these biomarkers could itself indicate pathology. Furthermore, the ratio between regional retinal and hippocampal thickness correlates with cognitive function in patients with MCI. We therefore demonstrate that the combination of biological markers derived from MRI and OCT reveals more information about the neurodegenerative process in patients with early cognitive decline compared with considering the markers separately.

## CONFLICT OF INTEREST

Dr. Donix and Dr. Brandt received research support from the Roland Ernst Stiftung. Dr. Donix received research support from the Robert Pfleger Stiftung. Dr. Linn reports speaker's honoria from Bayer Healthcare. Dr. Ziemssen received personal compensation from Biogen, Bayer, Celgene, Novartis, Roche, Sanofi, Teva for the consulting services and additional financial support for the research activities from Bayer, BAT; Biogen, Novartis, Teva, and Sanofi. The other authors report no financial relationships with commercial interests.

## AUTHOR CONTRIBUTIONS

Moritz Brandt designed the study; Dierk Wittig, Wiebke Hermann, Maren Dittmer, Annett Werner, Tjalf Ziemssen and Liane Jacobi acquired the data; Markus Donix, Dierk Wittig, Wiebke Hermann, Robert Haussmann, Franziska Bienert, Maria Buthut, Liane Jacobi and Moritz Brandt analyzed the data; Markus Donix and Moritz Brandt drafted the manuscript, Markus Donix, Moritz Brandt, Dierk Wittig, Wiebke Hermann, Maren Dittmer, Annett Werner, Tjalf Ziemssen, Jennifer Linn, Robert Haussmann, Franziska Bienert, Maria Buthut, and Liane Jacobi revised the manuscript for intellectual content.

## Data Availability

The data that support the findings of this study are available from the corresponding author upon reasonable request.
